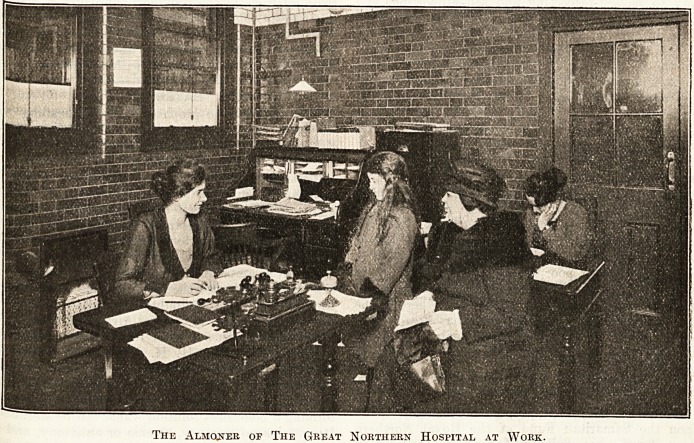# The Lady Almoner

**Published:** 1923-03

**Authors:** Joan Kennedy


### THE LADY ALMONER.

By JOAN KENNEDY.

THOUSANDS of patients pass through the doors of a great hospital every year, and not all are in-patients. Many receive advice and treatment which can be carried out at home, and in every hospital the out-patient department is very important. Long hours are devoted by skilled physicians and surgeons to the treatment of out-patients, but having given their help, what is impossible to the medical men is to secure that the treatment is not rendered inadequate for want of help and care in carrying out their instructions.

THE ALMONER OF THE GREAT NORTHERN HOSPITAL AT WORK.

### ENTER THE GOOD FAIRY.

It is here that the Hospital Almoner comes in. The other day I chatted with the Lady Almoner at the Royal Northern Hospital in the Holloway Road, and gained some idea of the importance and variety of this form of social service. It is estimated that an average of thirty-five new out-patients and thirty old cases a day must be seen by the Lady Almoner. This does not, of course, comprise the total number of daily attendances, but at present, owing to the lack of space and funds, the Almoner's department cannot cope with the entire number. The out-patient department of the hospital was built to deal with 40,000 attendances per annum, but the attendances have now reached a yearly total of 180,000. "I might call myself a social maid of all work," she told me with a smile. And when I heard something of her duties, it seemed to me the expression was very apt. But multitudinous as those duties are, involving a wide knowledge of human nature, great tact and sympathy, the Almoner herself weeds them down to two fundamental principles—to help patients and to protect the hospital.

### THE ALMONER AS PROTECTRESS.

Hospitals are not only used, but occasionally abused. The Almoner must sift out cases unsuitable for hospital treatment and see that private advice is secured when the patient is well able to afford it. In this way she protects the hospital. But, important though this part of her work be, the largest part is of a constructive nature. It is she who sees that the patients benefit to the full by the skilled advice of the doctors, that they are assisted in obtaining those forms of material relief, such as surgical appliances, convalescent treatment, necessary extra nourishment, and so on, which the doctor may order. The right centres for relief are approached. The patient is helped to make use of the facilities offered, while the hospital is protected.

### MANY ACTIVITIES.

At the Royal Northern Hospital the Lady Almoner makes her call for financial aid upon the Samaritan Fund of the Ladies' Association, the Charity Organisation Society, the Invalid Children's Aid Association, the Hospital Saturday Fund and the Hospital Sunday Fund, the Guardians, the Borough of Islington Charities Association, and others too numerous to name, while in many cases she will interview the employer of the patient and obtain from him help towards the recovery of his employé. In scores of ways she helps the patient, sometimes being instrumental in getting his job kept open until recovery enables him to return, and often helping him to suitable work after an illness which may have rendered it impossible for him to resume the old. There is no limit to her duties, which form social service of the highest order.

### The Helping Hand.

Among the great populace of a million served by the Royal Northern are many who are unable to afford to pay for the help they receive at the hospital; others would gladly pay back instalments when they are again earning. Many are helped in this way to be self-respecting and self-supporting, and gladly pay back to the fund which has befriended them in time of great need. A man loses his leg. To get an artificial one is beyond his purse. Without the artificial limb he is a cripple; with it he can go back to work. Funds from every available source are raised in small sums by the Lady Almoner, the leg is provided, and in many cases the man repays his share when he returns to work. Children require surgical boots; there are needed elastic bandages, glass eyes, artificial teeth, supports for flat feet, trusses, belts, spinal apparatus, and all kinds of surgical appliances beyond the reach of the poor unless they are helped. In other cases better food is necessary to help along the treatment, and the little luxuries could never be procured by the patient without help. Even coal has had to be provided in some instances in poor homes. Then, again, there is that visit to the convalescent home which would mean everything to the invalid. But where is the money to come from, when a patient has already been out of work for weeks? Here, again, the Lady Almoner paves the way with help secured from many agencies.

### A Maid of All Work.

From the last annual report of the calls made upon the Samaritan Fund at the Royal Northern these figures are culled:—Patients sent to convalescent home, 385; number of fares paid, 26; surgical appliances supplied, 324; assistance to patients with milk, coal, &c., 25. Each case must be investigated by the Almoner. It is she who helps in rescue work by her practical sympathy and help at the right time, she who arranges that the poor home may be run by another while the worn mother comes into hospital, she who finds long-lost friends for lonely sufferers, and generally works with the doctor in doing that side of the treatment which is as important as medicine. No wonder we may call the Lady Almoner the social maid of all work.

### The Word in Season.

Just to illustrate the variety of her duties, let me quote two little stories that were told to me. A woman had recovered from a serious operation in hospital, but nobody could understand why she did not appear to pull up when she was receiving after-treatment. The Lady Almoner's tactful sympathy found out that she was worried, and all because her husband played dominoes every Saturday afternoon instead of taking an interest in his home. This apparently trivial fact was retarding her treatment, because, in her lowered state of health and vitality, she allowed it to assume gigantic significance. Mr. Husband was interviewed by the Almoner. Very tactfully she suggested that he should take his wife and children to "the pictures" sometimes on a Saturday afternoon. He acted on the suggestion, and his wife soon began to regain her health. Then there was Miss R., a nurse who was in hospital with heart disease. She could never resume her duties as a nurse, and seemed to have no idea of where to find relations. There was a cousin somewhere in London in comfortable circumstances and there were relations in the provinces, but Miss R. had no addresses. The Almoner took up the case, and after a long search, found the London cousin. She was able to help and willing to do so, and through her the nurse was safely placed with her relatives.

### "BAYER 205."

It is fairly safe to prophesy that this heading will soon become as familiar as "Ehrlich 606." "Bayer 205" is a drug prepared by the firm of F. Bayer & Co., Leverkusen, Cologne, and the importance attached to its efficacy in a variety of tropical diseases due to trypanosomes is so great that it has been said (and stoutly denied), that hopes are entertained in Germany that by keeping its composition and the method of its manufacture secret, it may be used as a pawn in Germany's campaign for recovering her colonies. Of the prophylactic and therapeutic value of the drug there seems to be little doubt, and it may conceivably prove to be the key to tropical Africa and other parts of the world with a sinister reputation for trypanosome diseases. Dr. H. H. Dale, of the National Institute for Medical Research, has already given an account of certain of the properties of the drug, which is a white powder freely soluble in water, forming a colourless solution which can be sterilised. It contains no mercury, arsenic or antimony, and it belongs to a group of organic compounds capable of an almost infinite variety of modifications and improvements. Hitherto the chief objection to drugs used for the control of trypanosome diseases, such as sleeping sickness, has been that the dose required to destroy the infecting organisms was perilously close to a dose calculated to injure their host. The therapeutic index of "Bayer 205" is 1 to 160, which means that the organisms can be destroyed in the body by a dose incapable of injuring it, and as it is retained in the body for months, it is not necessary, and indeed it is unwise, to repeat its administration frequently. It is effective against every one of the large family of trypanosomes, but it appears to be useless in tropical diseases due to other organisms than trypanosomes, and as a panacea for most microbic diseases it is likely to prove disappointing.

---

The Report of the Proceedings of the National Milk Conference, held at the Guildhall last October, has been published by the National Clean Milk Society on behalf of the Conference Committee, 3 Bedford Square, W.C.1, at 3s. post free.

## Figures and Tables

**Figure f1:**